# Tweeting the Meeting: An In-Depth Analysis of Twitter Activity at Kidney Week 2011

**DOI:** 10.1371/journal.pone.0040253

**Published:** 2012-07-05

**Authors:** Tejas Desai, Afreen Shariff, Aabid Shariff, Mark Kats, Xiangming Fang, Cynthia Christiano, Maria Ferris

**Affiliations:** 1 Division of Nephrology and Hypertension, East Carolina University, Greenville, North Carolina, United States of America; 2 Department of Internal Medicine, East Carolina University, Greenville, North Carolina, United States of America; 3 Lane Center for Computational Biology, Carnegie Mellon University, Pittsburgh, Pennsylvania, United States of America; 4 Department of Nephrology, Northeast Georgia Diagnostic Clinic, Gainesville, Georgia, United States of America; 5 Department of Biostatistics, East Carolina University, Greenville, North Carolina, United States of America; 6 Division of Nephrology and Hypertension, University of North Carolina, Chapel Hill, North Carolina, United States of America; University of Pittsburgh Medical Center, United States of America

## Abstract

In recent years, the American Society of Nephrology (ASN) has increased its efforts to use its annual conference to inform and educate the public about kidney disease. Social media, including Twitter, has been one method used by the Society to accomplish this goal. Twitter is a popular microblogging service that serves as a potent tool for disseminating information. It allows for short messages (140 characters) to be composed by any author and distributes those messages globally and quickly. The dissemination of information is necessary if Twitter is to be considered a tool that can increase public awareness of kidney disease. We hypothesized that content, citation, and sentiment analyses of tweets generated from Kidney Week 2011 would reveal a large number of educational tweets that were disseminated to the public. An ideal tweet for accomplishing this goal would include three key features: 1) informative content, 2) internal citations, and 3) positive sentiment score. Informative content was found in 29% of messages, greater than that found in a similarly sized medical conference (2011 ADA Conference, 16%). Informative tweets were more likely to be internally, rather than externally, cited (38% versus 22%, p<0.0001), thereby amplifying the original information to an even larger audience. Informative tweets had more negative sentiment scores than uninformative tweets (means −0.162 versus 0.199 respectively, p<0.0001), therefore amplifying a tweet whose content had a negative tone. Our investigation highlights significant areas of promise and improvement in using Twitter to disseminate medical information in nephrology from a scientific conference. This goal is pertinent to many nephrology-focused conferences that wish to increase public awareness of kidney disease.

## Introduction

Each year the American Society of Nephrology (ASN) holds its annual scientific conference, Kidney Week, where more than 10,000 national and international healthcare providers (physicians, advanced practitioners, nurses, technicians, trainees, and pharmaceutical representatives) discuss advances in research and new scientific breakthroughs. In recent years, the ASN has increased its efforts to use these annual conferences to inform and educate the public about kidney disease. This focus is a result of the significant lack of understanding about and recognition of kidney disease amongst the population. In his opening plenary address for Kidney Week 2011, former ASN President Dr. Joseph Bonventre indicated that public awareness and education are important challenges for the Society in the coming years [1]. Indeed, the first step in increasing public awareness of kidney disease is to identify and utilize a communication method that successfully generates and spreads educational information. Not surprisingly, social media has been viewed by the ASN, as well as other societies, as a potentially useful communication tool [1], [2]. Since 2009, scientific and educational societies have used Twitter to spread information regarding their respective missions, using one of three analyses [3]–[6]. Indeed Twitter can disseminate non-medical information effectively and healthcare providers have used Twitter to share medical information as well [7]–[10]. We hypothesized that content, citation, and sentiment analyses of tweets generated from Kidney Week 2011 would reveal a large number of educational tweets that were disseminated to the public.

## Methods

Kidney Week 2011 was held from 8–13 November 2011. The conference was subdivided into pre-courses (days −2 and −1) and full conference (days 0to +3). Day 0 was defined as 10 November 2011. Kidney Week 2011 was open to healthcare providers of all training/education levels. Educational sessions/posters were not subdivided by educational tracks but rather categorized into one of 12 learning pathways for all conference attendees.

Tweets deposited in the official Kidney Week 2011 public timeline were analyzed. This timeline was established and promoted by the ASN to collect tweets generated from or about the conference from any Twitter account holder. The timeline was freely available to anyone searching *#kidneywk11* regardless of Twitter or ASN membership status, and the collected tweets are freely available upon request with the corresponding author TD [11], [12]. Tweets were de-identified in accordance with established guidelines regarding personally identifiable information (PII) through social media [13], [14]. We presumed that tweeters using *#kidneywk11* took part in the conference.

Content analysis was performed by classifying each tweet as informative or uninformative, using an industry-standard classification system [15]. Informative tweets were defined as those that educated the reader about any aspect of kidney disease. Uninformative tweets were defined as those that did not educate the reader and were further subdivided into: 1) advertisement, 2) status update, 3) query, 4) direct message, 5) opinion, or 6) other [15]. In addition, each tweet was categorized into one of twelve official, pre-established Kidney Week 2011 learning pathways, by cross-referencing the keyword(s) in each tweet with the Kidney Week 2011 Program Builder [16]. If a learning pathway could not be ascribed, the tweet was considered uncategorized.

Citation analysis was performed by classifying each tweet as having an internal, external, or no citation, using a pre-defined, Twitter-specific classification system [3], [17]. Tweets with internal citations, commonly referred to as retweets, contained the prefix RT@ while external citations contained shortened universal resource locators (URLs) to third party websites (Table 1) [3].

**Table 1:** Definitions.

Term (in order of appearance in the body of the text)	Definition
Twitter*	A service for friends, family, and co–workers to communicate and stay connected through the exchange of quick, frequent messages
Microblog**	A broadcast medium in the form of blogging. A microblog differs from a traditional blog in that its content is typically smaller in both actual and aggregate file size. Microblogs allow users to exchange small elements of content such as short sentences, individual images, or video links
Tweet*	A message composed on Twitter of 140 characters or less
Re-tweet*	A re-distribution of someone else’s tweet. Re-tweeted messages are designated by the prefix “RT” and amplify the content within the tweet
Timeline*	A collected stream of Tweets listed in real-time order
“@” symbol	The “@” is used to signify when a particular post is addressed to or references someone.
“#” symbol*	Called a hashtag, this symbol is used to mark keywords or topics in a Tweet. It is a way to categorize messages
Tweeter	One who composes messages using Twitter

* As defined by Twitter (http://support.twitter.com).

** As defined by Wikipedia (http://www.wikipedia.com).

Sentiment scores were calculated by performing conventional linguistic analyses of each tweet using a modified Affective Norms for English Words sentiment lexicon (AFINN) [18]–[21]. The AFINN lexicon was developed specifically for the text of microblogs such as Twitter [18]. Each word in the lexicon is given a dimensionless integer value from −5 (highly negative) to +5 (highly positive). We ascribed these values from the lexicon to each word in a tweet using an automated complier and averaged the values to obtain a sentiment score for that tweet.

The ideal tweet was pre-defined as one that had the greatest ability to disseminate educational information about kidney disease because it was 1) informative, 2) internally cited and, 3) had a positive sentiment score. TD, AS, and MK independently classified each tweet and met as a committee to resolve classification differences (Table 2). Two proportion z and Chi-square tests were performed on data in the content analysis. Data in the citation analysis underwent two proportion z testing, while data in the sentiment analysis underwent t testing and ANOVA. The investigation was conducted from 1 June to 14 November 2011 and was approved by the East Carolina University Institutional Review Board.

**Table 2:** Representative examples of tweets generated during Kidney Week 2011.

Type of Tweet	Representative Example
Content: Informative	Fruit and veggie supplements can be as effective as bicarbonate at reducing acid generation and loss of GFR. #kidneywk11
Content: Uninformative – Advertisement	Still time to pick up a free copy and discount subscription to JAMA and/or Archives of Internal Medicine, booth 4*** #kidneywk11
Content: Uninformative – Status Update	Readying ourselves for Kidney Prom, AKA Presidents's Dinner. #kidneywk11 (@ Philadelphia Marriott Downtown) http://t.co/KML3d
Content: Uninformative – Opinion	Unfortunately nothing new so far this year at the asn. #kidneywk11 #kidney
Content: Uninformative – Query	What sessions are students going to this morning? #kidwkstu #kidneywk11
Content: Uninformative – Direct Message	@ *** @*** Don't Miss! Nephrology Education Research. Fri. 4:30–6:30 room 112 #kidneywk11 #Kidwkstu
Sentiment: Positive	@ *** agree- RTA session 4:30–6:30 rm 113 looks good. I will b there! Excellent for students/res #kidneywk11 #kidwkstu
Sentiment: Less Positive	No photography allowed in the poster sessions at #kidneywk11 ? WTF!
Sentiment: Neutral	@ ***: Call-ins to press briefing - please no talking! Coming through. #kidneywk11
Citation: Internal	RT @***: Students and residents meeting for guided poster tour- gathering near the Fresenius booth. Join us! #kidwkstu #kidneywk11
Citation: External	Press: >15% of kidney disease pts take herbs or supplements that NKF sz poss harmful to health http://t.co/9Px7l #kidneywk11
Citation: Both	RT @***: Press brief 9:30 am TODAY Rm 303A Impact of Diet and Dialysis on Kidney Pts CALL-IN INFO: http://t.co/fWgTq #kidneywk11
Citation: None	Don't berate yourself if you can't normalize your body weight. You're fighting powerful biological systems. #kidneywk11

*** Denotes de-identified (total or partial) username or URL.

## Results

**Figure 1:**
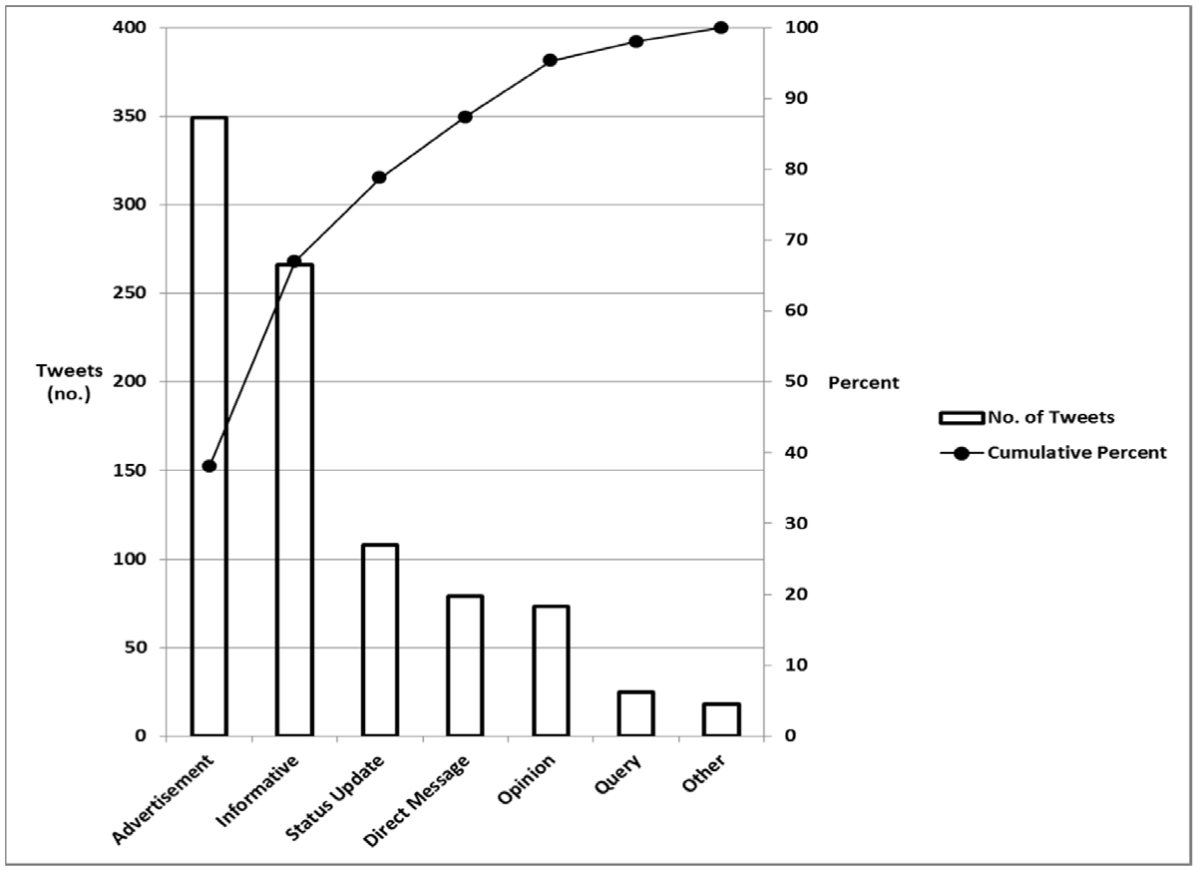
Tweets from Kidney Week 2011.

A total of 172 individuals composed at least one tweet (1.4% of the total number of conference attendees) for a total of 993 tweets (917 English; 76 Spanish). There were 2.5 times more uninformative than informative tweets (651 versus 266, respectively; p<0.0001). Approximately 80% of all tweets were advertisements (38%), informative (29%), and opinion (12%) (p<0.0001 between pairs) (Figure 1).

**Figure 2:**
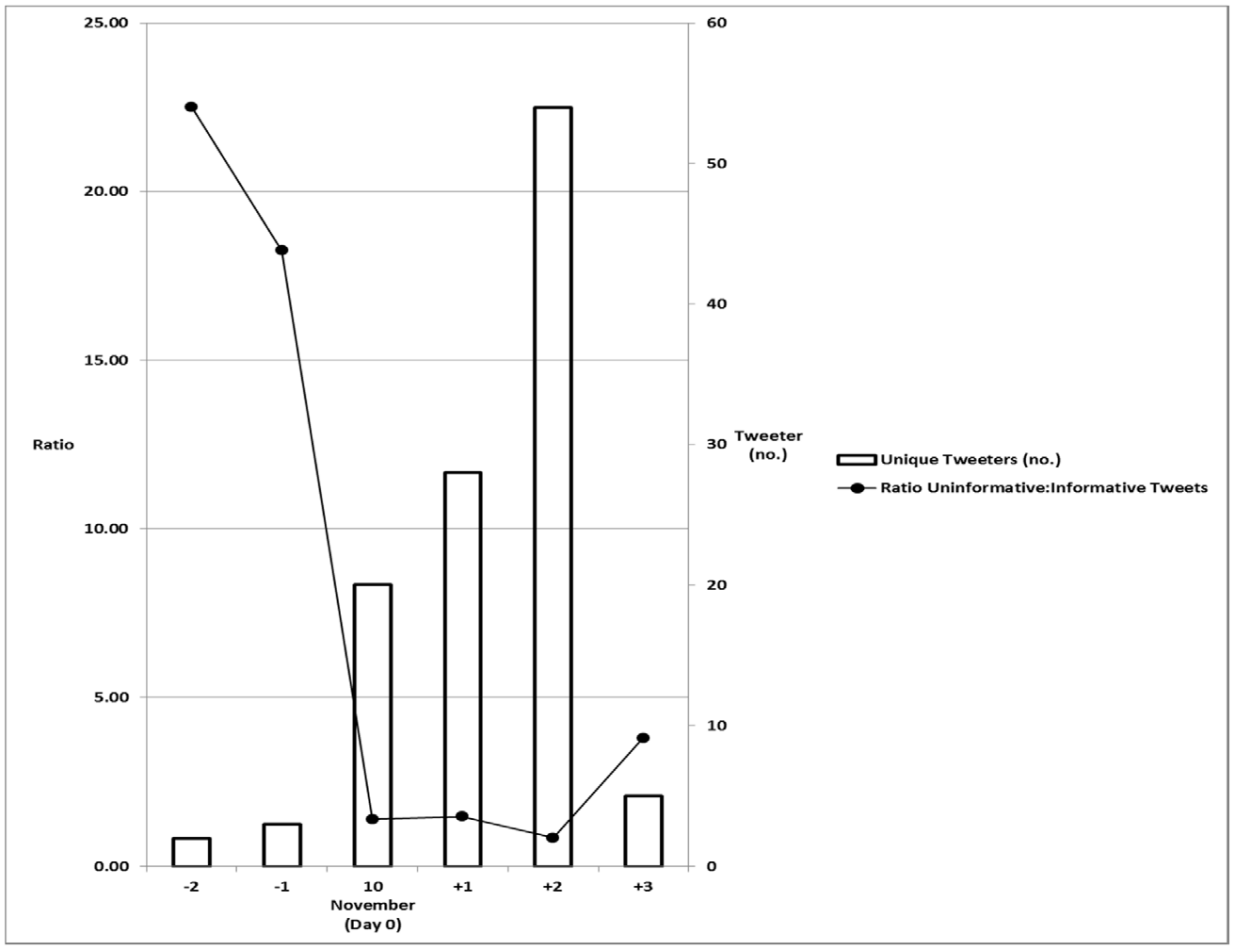
Relationship between numbers of tweets and tweeters.

Over 99% of informative and 70% of uninformative tweets were composed from days −2 to +3 of the conference. In the intra-session periods, the ratio of uninformative to informative tweets was 1.5. This ratio increased to 3.9 in the inter-session periods (p<0.0001). The conference days with the lowest number of informative tweets and unique authors (tweeters) were days −2 and −1 (pre-course sessions), but there was no significant relation between these 2 variables (r = 0.68, p = 0.137) (Figure 2).

**Figure 3:**
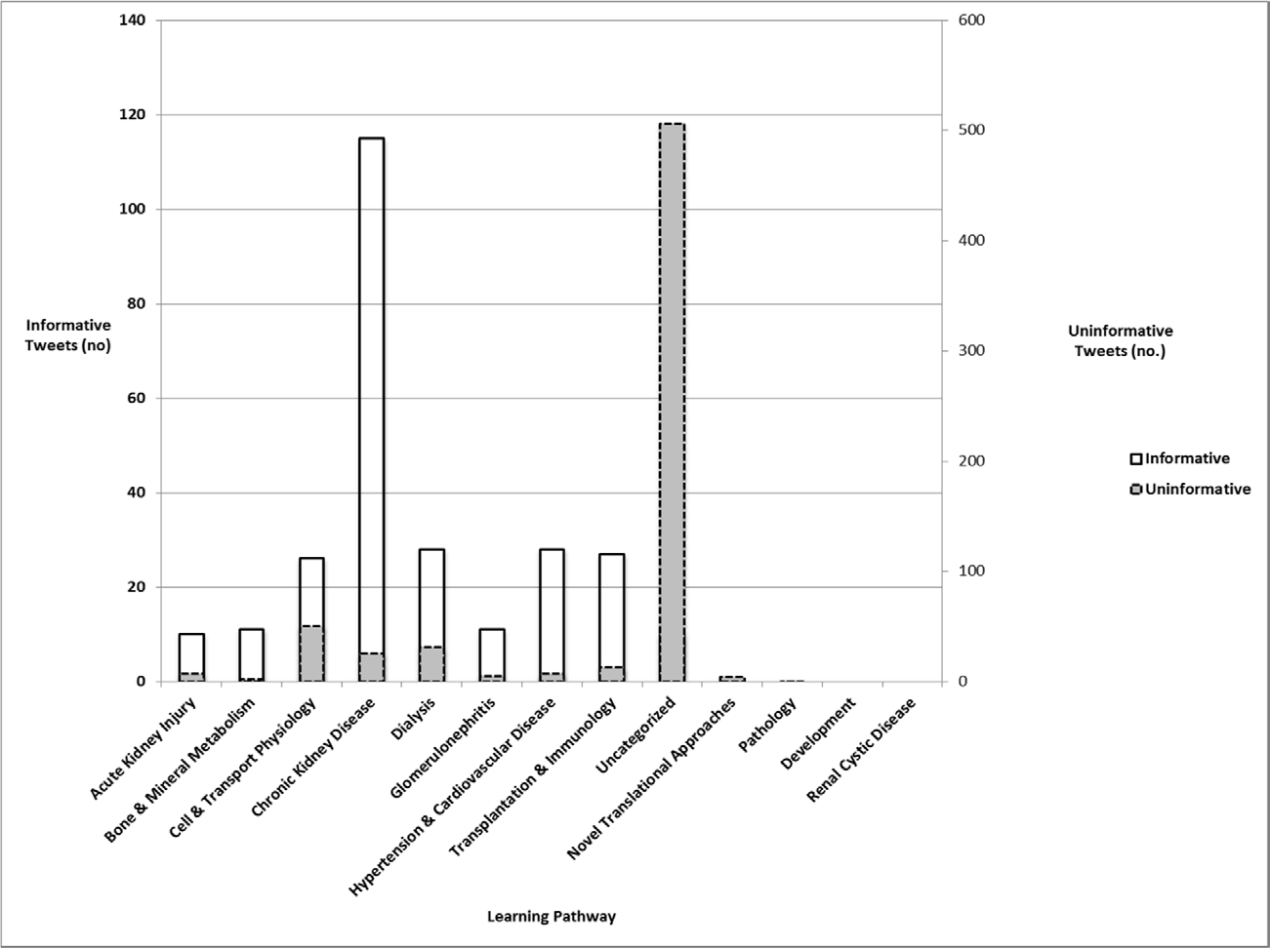
Distribution of tweet content.

Chronic kidney disease was the most popular learning pathway tweeted (Figure 3). Pathology, Development, Renal Cystic Disease, and Novel Translational Approaches generated zero informative tweets despite 539 total sessions or posters pertaining to these pathways. The remaining 8 learning pathways showed a significant correlation between the number of informative tweets and number of sessions or posters in each pathway (r = 0.82, p = 0.001) (Table 3). A scatter plot (not shown) suggested a linear relationship these 2 variables. Subsequent regression analysis revealed that approximately 250 sessions or posters per pathway were needed to generate one informative tweet (informative tweets = 15+0.109 x number of sessions/posters; p = 0.001).

**Table 3:** Actual and Predicted Informative Tweets.

Learning Pathway	Actual InformativeTweets (No.)	Predicted No. of InformativeTweets (95% CI)	Sessions or Posters (No.)
Acute Kidney Injury	10	25.14 (12.80, 37.47)	369
Bone & Mineral Metabolism	11	2.10 (−0.91, 25.10)	249
Cell & Transport Physiology	26	14.05 (1.35, 26.76)	267
Chronic Kidney Disease	115	82.08 (50.00, 114.15)	893
Dialysis	28	54.58 (34.28, 74.89)	640
Glomerulonephritis	11	40.57 (25.17, 55.96)	511
Hypertension & Cardiovascular Disease	28	5.36 (−9.12, 19.84)	187
Transplantation & Immunology	27	23.40 (11.16, 35.64)	353
Uncategorized	10	Not available	N/A
Novel Translational Approaches	0	−4.85 (−22.53, 12.82)	93
Pathology	0	2.75 (−12.45, 17.95)	163
Development	0	0.25 (−15.71, 16.21)	140
Renal Cystic Disease	0	0.58 (−15.28, 16.44)	143

**Figure 4:**
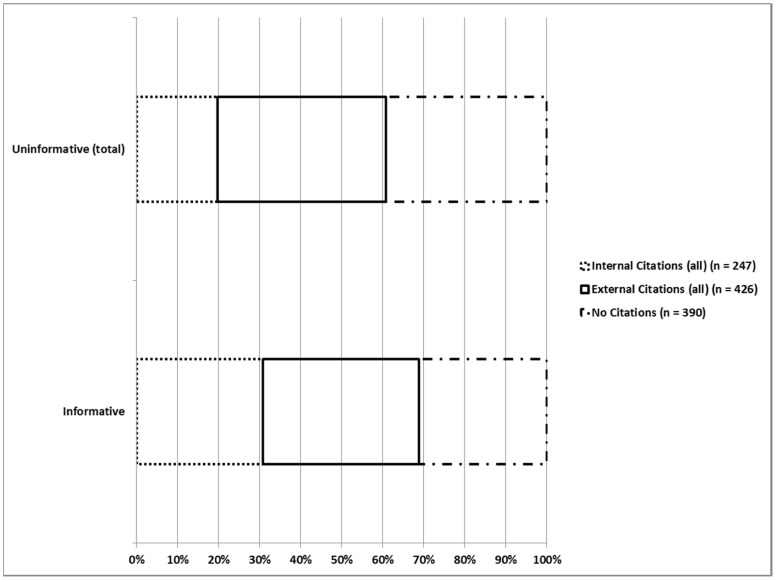
Distribution of tweet citations.

**Figure 5:**
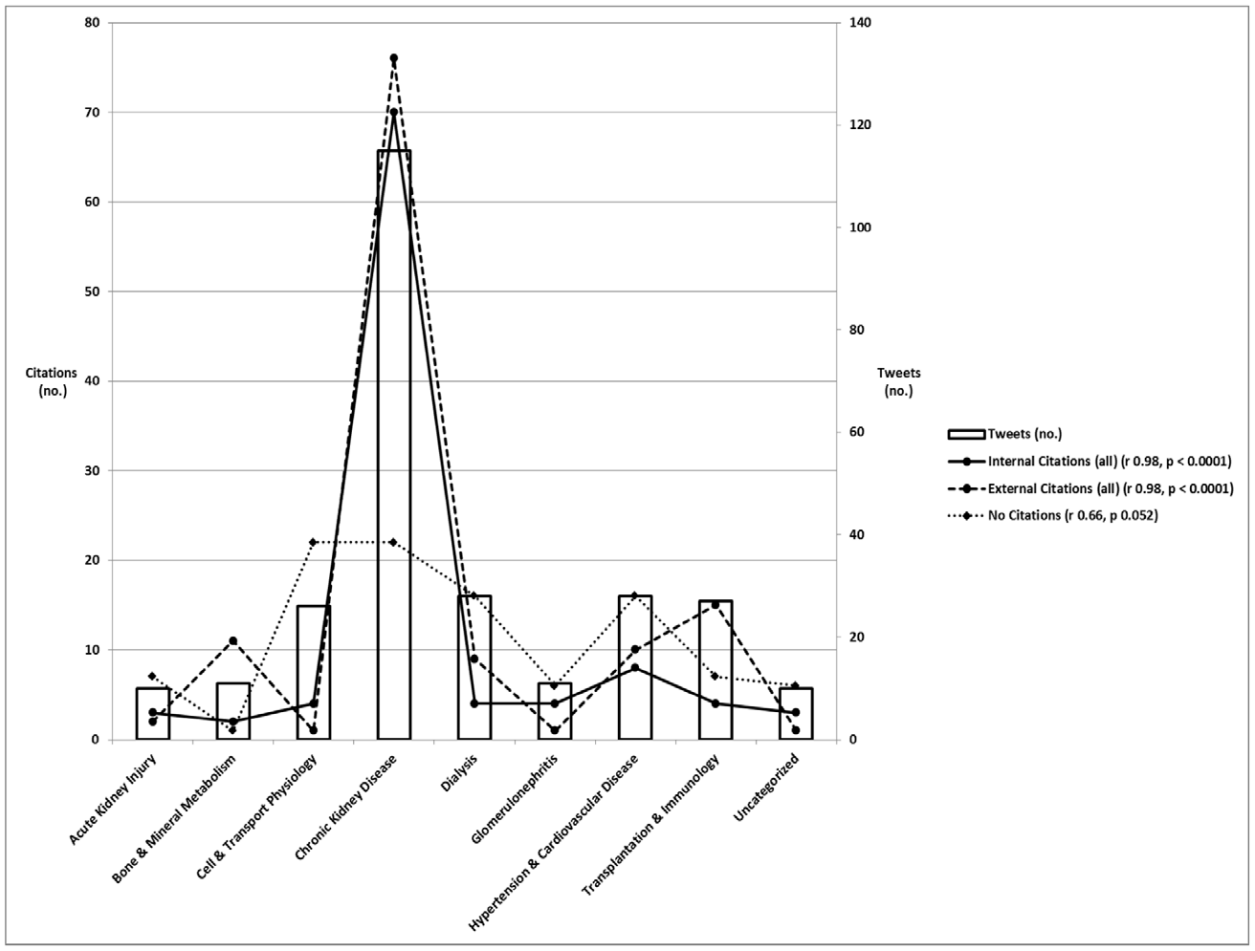
Relationships between numbers of tweets and citations.

Less than 50% of all tweets contained no citations (p<0.0001). The remaining 57% of tweets contained more external than internal citations (426 versus 247 respectively, p<0.0001). Among external citations, 70% were uninformative and 30% informative (p<0.0001) (Table 4). The largest contribution to externally cited uninformative tweets was advertisements at 75%. There were approximately 1.4 times as many internally cited uninformative tweets than similarly cited informative tweets (145 versus 102 respectively; p = 0.007), and informative tweets were more likely to be internally cited than uninformative tweets (38% versus 22%, p<0.0001) (Figure 4). Moreover, the number of internally cited informative tweets correlated with the number of all such tweets in 8 learning pathways (r = 0.98, p<0.0001) (Figure 5).

**Table 4:** Citations of tweets.

	Internal Citations (all)* (n = 247)	External Citations (all)* (n = 426)	No Citations (n = 390)
Informative	102	126	103
Uninformative (total)	145	300	287
Advertisement	94	226	92
Status Update	17	31	68
Direct Message	18	27	40
Query	5	1	19
Opinion	10	9	57
Other	1	6	11

* Total citations (1063) greater than total number of tweets (917) because some messages contained both internal and external citations and are represented more than once.

The mean sentiment score for all tweets was 0.094 (SD 0.476; range −1.70, 2.67). Informative tweets had more negative sentiment scores than uninformative tweets (means −0.162 versus 0.199 respectively, p<0.0001). Opinion tweets had the highest mean score (0.454) amongst all types of tweets. There were no statistical differences between the mean scores of informative tweets based on learning pathway.

**Figure 6:**
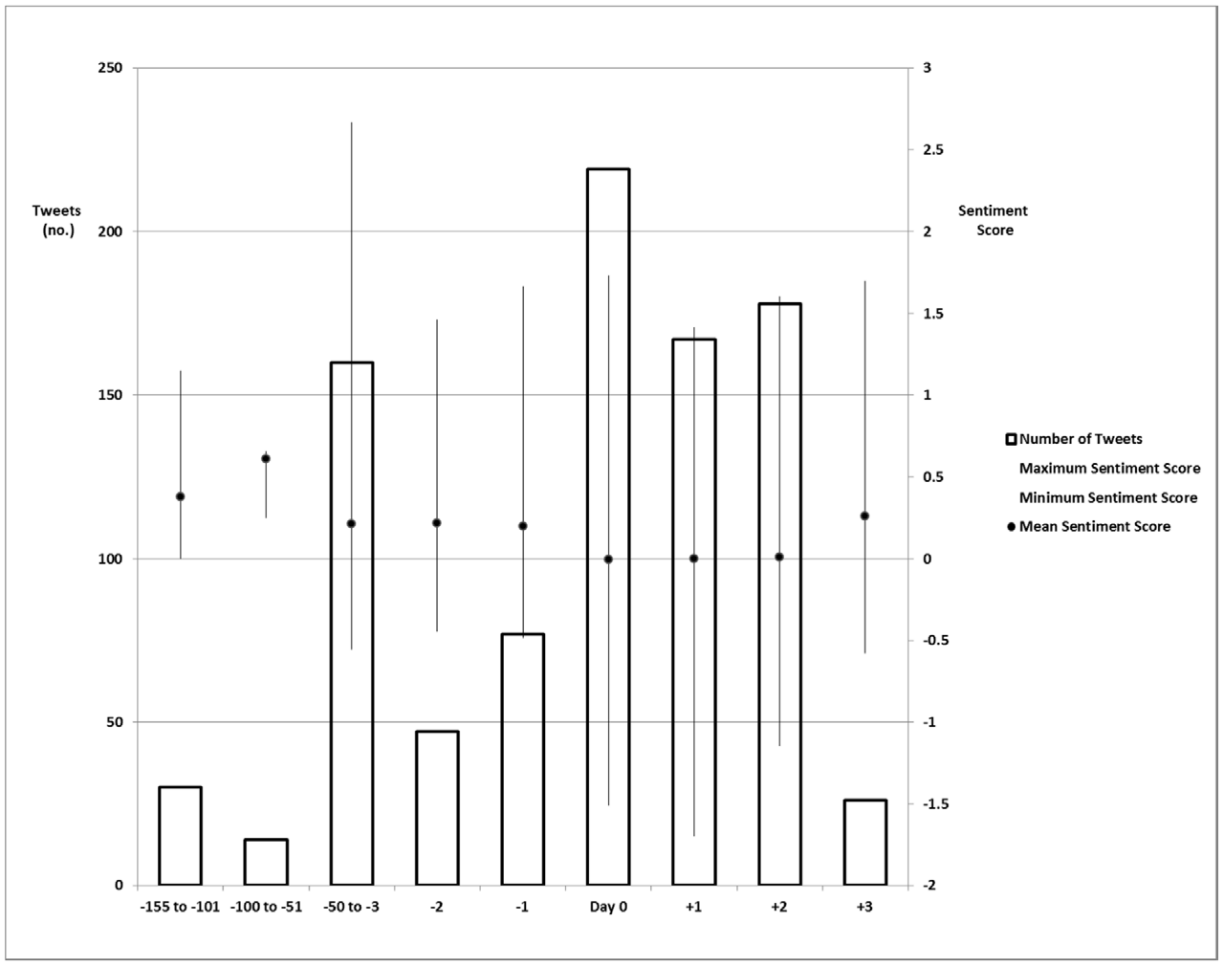
Distribution of sentiment scores.

Tweets without citations had the most positive sentiment score while tweets with both citations (internal and external) had the most negative score (means 0.166 versus −0.070 respectively, p = 0.0001). Tweets that were exclusively internally or externally cited had positive scores (means 0.125 and 0.680 respectively, p = 0.407). The most positive tweets were composed from days −100 to −51 (mean 0.610, SD 0.105). There was decline in mean sentiment score of all tweets leading up to the start of Kidney Week 2011 (Figure 6). The sentiment score from days −155 to −3 was more positive than during days −2 to +3 (means 0.264 versus 0.045, respectively, p<0.0001).

## Discussion

Twitter can best be used to disseminate educational information about kidney disease if tweets have 3 key features: informative content, internal citations, and a positive sentiment score. Informative content improves the reader’s understanding of kidney disease. Internal citations allow the content of the tweet to be amplified, thereby reaching the largest number of readers [22]. Positive scores indicate the mood of the tweeter and his/her impression of the conference [18]–[22]. A positive score tweet leaves a good impression with the reader and increases the likelihood that future tweets will be amplified by that reader. Thus, the challenges set forth by the ASN can be fulfilled if these features are prevalent among the tweets of this and future Kidney Week meetings.

Few conference attendees composed tweets (1.4%). This may be due to unfamiliarity with Twitter or that the Kidney Week 2011 timeline (*#kidneywk11*) was 1) unknown to or 2) misspelled by a large number of attendees [22]. Nevertheless, 29% of all tweets were informative, greater than that found in a similarly sized medical conference (2011 American Diabetes Association meeting, 14%) [6]. Although *#kidneywk11* was established in June 2011, only 1% of informative tweets were composed prior to the pre-courses (day −2). Therefore, readers were underexposed to topics about kidney disease in the months leading up to Kidney Week 2011. Comparable scientific conferences have created a more steady increase in tweet number leading up to their respective conferences, but have not published specific strategies to avoid the spike that occurred in *#kidneywk11* [4], [6]. There is a need for more informative tweets to be composed from days −155 to −3 in order to maintain a steady flow of informative information. One suggestion to accomplish this goal is to highlight key information from previous years’ conferences and/or provide background information of new data that will be presented.

There were zero informative tweets pertaining to a third of learning pathways. This finding highlights a lost opportunity to share information with the public of issues germane to those pathways. Although this investigation does not establish a causal relationship, there is a correlation between the number of informative tweets and sessions/posters that should not be ignored. Prospectively identifying learning pathways with less than 250 sessions and/or posters will help conference organizers recognize those pathways that are likely to be underrepresented in the Twitter timeline. Conference organizers can then selectively focus on these pathways to increase the spread of educational information in these topics.

Tweet amplification (internal citations) is a critical component to disseminating educational information. Data from a random sample of 720,000 tweets suggest a baseline amplification percentage of only 3% [5]. Although 38% of all informative tweets from Kidney Week 2011 were amplified, this percentage is less than that seen in the 2011 American Diabetes Association meetings (47%) [6]. Not surprisingly, the total number of uninformative tweets that were amplified was greater than amplified informative tweets, but the ratio was more favorable than the total number of tweets (1.4∶1 amplified uninformative to amplified informative versus 2.5∶1 uninformative to informative). In addition, there was a more even distribution of amplified tweets across all citation groups. These findings not only suggest that there was a healthy amount of informative tweet amplification, but that Twitter use distributed educational tweets to the largest possible audience. More importantly, the mechanics of tweet amplification, including tweeter knowledge and willingness to internally cite tweets, was already in place at Kidney Week 2011. Indeed all that would be required to increase tweet amplification is to increase the number of informative tweets generated.

In its totality, *#kidneywk11* tweets conveyed a slightly positive sentiment. Specifically, informative messages had the second highest positive sentiment scores. It is important to note that a positive sentiment score is not a prerequisite for message amplification (internal citation). Moreover, the absolute score of each tweet has not been shown to correlate with the educational value of the content within that tweet. The key finding in the sentiment analysis was the trend of scores. Tweets were more negative during (days −2 to +3) than leading up to (days −155 to −3) the conference. We were surprised that tweets adopted a more negative tone approaching the start of the conference. One explanation could be the larger number of tweets from days 0–2 than any other time period. A second explanation could be that tweets from days 0 to +2 contained more debates and differing viewpoints. However, since the public is unable to group tweets in *#kidneywk11* by conversations, there is a real potential that the reader walked away with a negative perception of Kidney Week 2011. This negative perception could limit the dissemination of future kidney-specific educational information. Unfortunately, scientific conferences have not published sentiment data and trends to allow for meaningful comparisons [4]–[6]. As a result, investigations are now needed to elucidate 1) reader perceptions when sentiment scores are low, 2) the challenges of improving tweet sentiment scores and 3) effective remedies when scores are low.

There are an additional 6 notable points of this investigation. First, tweets that were not deposited in the *#kidneywk11* timeline were not analyzed in this investigation. However, this deficiency is unlikely to significantly alter our results because *#kidneywk11* was the official Twitter timeline for Kidney Week 2011.

Second, we calculated sentiment scores through a lexicon-based linguistic analysis of each tweet. While this method is well accepted and validated, it may not encompass the full range of emotions that a “bag of words” model could capture [18]–[21]. Moreover, the lexicon used was comprised of English words only, and thus non-English tweets (76 out of 993) were excluded from the analyses. We did not translate the non-English tweets; doing so could have resulted in an unintended change of tone and thereby skew the results.

Third, we correlated the number of sessions and posters with the number of informative tweets. A more meaningful correlation might be the time exposed to each learning pathway with the number of informative tweets, but the former could not be accurately measured or calculated.

Fourth, we did not compare tweets from similarly sized nephrology or other medical conferences. Our comparison was limited between tweets from Kidney Week 2011 and the 2011 ADA meetings because conference officials for both had documented an interest in using Twitter to increase public awareness [1], [6]. A comparison with other nephrology or medical conferences would have been more meaningful had those respective conference officials/organizers documented a similar intention.

Fifth, we analyzed 917 tweets from 172 unique tweeters. While these numbers might be considered small, they represent the entire population of 1) English-worded tweets and 2) tweeters from the meeting. In addition, this study had an 80% power to detect statistically significant differences.

Finally, a large percentage of tweets were advertisements. This finding makes the use of Twitter as a communication tool challenging as advertisers can misuse it under the disguise of education.

The tweets analyzed are part of the public domain. Neither the ASN, Twitter, nor the individual tweeter own any or all tweets deposited in *#kidneywk11* [23]–[24]. Tweets, by definition, are short expressions and are not protected under Section 102 of the US Copyright Act [25]. To the best of our knowledge, this report conforms to the Strengthening the Reporting of Observational Studies in Epidemiology (STROBE) guidelines [26].

Our investigation highlights significant areas of promise when using Twitter to share kidney-specific educational information with the public. In fact, our analyses are pertinent to any medical conference in which the organizers or healthcare providers wish to disseminate information to the general public. Dissemination of information is needed in order for the nephrology community to increase public awareness of kidney disease. This investigation focuses on how well Twitter was used to disseminate educational information from a scientific conference. In the future, investigations are needed to determine if greater dissemination of educational information will lead to greater public awareness.
